# A Prospective Study of Brucellosis in Children: Relative Frequency of Pancytopenia

**DOI:** 10.4084/MJHID.2013.011

**Published:** 2013-02-16

**Authors:** Mohamed A El-Koumi, Mona Afify, Salha H Al-Zahrani

**Affiliations:** 1 Department of Pediatrics, Al-Khafji Joint Operation Hospital. Kingdom of Saudi Arabia.; 1 Department of Pediatrics, Zagazig University Hospital, Egypt.; 2 Department of Biology, Science College for Girls King AbdulAziz University. Kingdom of Saudi Arabia.

## Abstract

Hematological complications in brucellosis are common. Pancytopenia, although mainly reported in adults has also been described in children with brucellosis. This investigation was conducted to estimate the relative frequency of pancytopenia in children with brucellosis. The current study was conducted in Al-Khafji joint operation hospital, Saudi Arabia. Sixty patients with brucellosis, were enrolled in the study. Complete blood count (CBC) and blood culture were performed for all cases. Bone marrow (BM) aspiration was considered only in those with pancytopenia. Out of 60 children with brucellosis, 50 (83%) ingested raw animal milk and 27 (45%) had a positive family history of brucellosis. The common presenting symptoms and signs included excessive sweating (68%), bone aches (62%), chills (55%), arthritis (32%), hepatomegaly (18%), and splenomegaly (15%). The main hematological manifestations included anemia (43%), leukopenia (38%) and leukocytosis (20%). Pancytopenia was detected in 11 patients (18%). Blood culture for Brucella was positive in 38% (23 patients). B.melitensis from 21 patients was cultured in vitro. Out of 9 BM aspirate cultures, 3 were positive for B. melitensis. Out of 11 patients with pancytopenia, 9 (82%) patients had bone aches and weakness, 7 (64%) patients had sweating and chills, and 6 (55%) patients had petechiea and purpura. Conclusion: The current study concludes that although pancytopenia is an uncommon complication of brucellosis in children, it does occur. Therefore, brucellosis should be considered in the differential diagnosis of pancytopenia in children, particularly in endemic areas such as Saudi Arabia.

## Introduction

Brucellosis, a primarily contagious disease of domestic animals, is caused by small, fastidious gram-negative coccobacilli of the genus *Brucella*. There are four important species pathogenic to humans; *B. melitensis*; found primarily in goats, sheep and camels; *B. abortus* in cows; *B. suis* in pigs; and *B. canis* in dogs. The Brucella species differ in degree of virulence and invasiveness, *B. melitensis* being the most invasive and produces the most severe disease while *B. abortus* is the least invasive.^1^ In Saudi Arabia, human infection with *B. melitensis* is commonly encountered (80%–100%), while, infection with *B. abortus* is less frequent. Infection with other species has not been reported.^2^ Humans are commonly infected through ingestion of raw milk, cheese or meat, or through direct contact with infected animals, products of conception or animal discharges (e.g., among shepherds, farmers and veterinarians), and through inhalation of infectious aerosols (e.g., by workers in abattoirs and microbiology laboratories).^3^ Human brucellosis can be an acute or a chronic febrile illness and presents with a variety of manifestations after an incubation period, which can vary from 1 to 6 weeks or several months. Brucellosis may be difficult to distinguish clinically from a number of other infections such as typhoid fever, tuberculosis, infective endocarditis, and acute rheumatic fever.^4^ The symptoms of acute illness are fever, chills, headache, muscle and joint pains, malaise, nausea, night sweats and loss of appetite persisting 3 to 6 weeks. Brucellosis shows multisystem involvement.^5^ The disease also produces a variety of nonspecific hematological abnormalities. The BM and the spleen are commonly involved, and such involvement may result in a hypo plastic pattern on the peripheral blood smear.^6^ Hematological complications of brucellosis are common and can be multifactorial due to the pathogen’s tropism for central (BM) and peripheral (spleen) organs of the reticulo endothelial system (RES). Changes in the hematological parameters are observed in most patients, but pancytopenia is rare.^6^ Hemo-phagocytosis, hypersplenism or granulomatous changes in the BM may be responsible for pancytopenia occurring during brucellosis. Additionally, BM involvement due to simultaneous presentation of malignant diseases with brucellosis rarely lead to pancytopenia.^7^

Incidence of pancytopenia is 2–14% among adult patients affected by brucellosis.^8^ Although the presentation of acute brucellosis with mesenteric lymphadenitis and pancytopenia is rare, it must be considered in patients in endemic areas.^9^ The aim of this study was to estimate the relative frequency of pancytopenia in Saudi children with brucellosis.

## Patients and Methods

This study was conducted at Al-Khafji Joint Operation Hospital, Saudi Arabia from August 2011 to October 2012. All children suffering from fever for more than 5 days, without clinically evident cause for fever, with symptoms suggestive of brucellosis such as weight loss, weakness, anorexia and polyarthalgia were screened for brucellosis by a rapid slide serum agglutination test using plasmatic stained febrile antigens reagent code number FA/018 for *B. abortus* and FA/020 for *B. melitensis*. If positive result was obtained, tube agglutination test was performed. Titer of 1/20 up to 1/360 was done for each serum to avoid prozone effects. Titer of 1/160 or above and rising antibody titer were considered to be positive. Of the positive cases, by slide agglutination test, a 10 ml of blood samples and/or bone marrow aspirates were obtained under complete aseptic procedures, inoculated and mixed on Hemoline Performance Diphasique, BioMerieux blood culture system and Oxoid signal blood culture system code: BC0100. The medium was designed to create pressure in the sealed bottle when organisms were growing. A positive result is signaled when the blood/broth mixture rises above the green locking sleeve of the growth indicator device. Positive growth was subcultured on blood, chocolate and *MacConkey’s* agar media, both aerobically in 5% CO2 atmosphere and anaerobically. Gram stain, oxidase, catalase, urease and other biochemical reactions were performed for identification of brucella species.

All children with positive tube agglutination test or positive blood or BM cultures were enrolled in the current study. Baseline data were collected including demographic data, documented family history of brucellosis, ingestion of raw milk, cheese or meat or contact with infected animals or their products, and history of hematological disorders. Besides, through clinical examination was performed.

Laboratory workup included CBC, erythrocyte sedimentation rate (ESR), and C-reactive protein (CRP). When indicated, coagulation profile including prothrombin time (PT), activated partial thromboplastin time (APTT) and plasma fibrinogen level were assayed. All investigations considered the established reference values in childhood.^10^ Pancytopenia was considered if age-corrected white blood cells, platelets count and hemoglobin values are low.^10^ In cases with bicytopenia, and pancytopenia, BM aspiration/biopsy was also considered. CBC repeat was performed in cases with cytopenia.^5^ Data entry and statistical analysis were performed by application of the Statistical Package for the Social Sciences (SPSS; IBM, Inc., NY, USA). P-values <0.05 were considered statistically significant.

## Results

One hundred thirty-three patients were screened for brucellosis, of these, 84 were positive by rapid slide test. None of the screened children with titer < 1: 160 had positive blood or BM culture for brucellosis. Sixty children, diagnosed as brucellosis whose titer ≥1:160 by tube agglutination method, were enrolled in this prospective study. Age of the enrolled children ranged between 5–16 years (Mean ± SD: 7.6 ± 1.8), of which 43 (71.7%) were males. Fifty patients (83.3%) gave a history of raw milk/dairy products consumption and 27 (45%) gave a positive family history of brucellosis. Excessive sweating was a complaint in 41 patients (68.3%), bone aches in 37 patients (61.7%), and chills in 33 (55%) patients. Nineteen patients (32%) had arthritis and/or arthralgia, 11 (18%) patients had hepatomegaly, 9 (15%) patients had splenomegaly while only 4 patients (7%) had hepatosplenomegaly (Table 1).

Table 2 summarizes the hematological manifestations, cultures and agglutination titers among the 60 children suffering brucellosis. Twenty-six patients (43.3%) had anemia, 23 (38.3%) had leukopenia, 12 (20%) had leukocytosis and 11 patients (18.3%) had pancytopenia. Among the 23 patients (38.3%) with positive blood culture; *B. melitensis* was isolated in 21 (35%) cases and *B. abortus* in only 2 (3.3%) cases. BM culture was conducted for 9 patients (15%). Of these patients, 3 (5%) were positive for *B. melitensis*. Thirty-eight patients (63.3%) had an agglutination titer of 1/160–1/320, and 22 (36.7%) displayed an agglutination titers of 1/320–1/640 or more. Out of all patients with brucellosis, 11 patients (18.3%) had pancytopenia at diagnosis of which 6 patients (55%) had petechiae, pupura and/or bleeding. The majority of patients with pancytopenia (72.7%) had an agglutination titer of 1/320–1/640 or more. Interestingly, blood culture was positive for *B. melitensis* in all patients with pancytopenia **(**Table 3).

## Discussion

Brucellosis is primarily an infectious disease of domestic animals that is transmissible to humans. The source of infection is likely to be fresh unpasteurized milk or milk products consumption, or via a direct contact with infected animal tissues.^11^ Although brucellosis has been controlled in many developed countries, it remains an important health problem in developing countries, particularly in Mediterranean region, Middle East and West Asian countries.^12^ Hematological complications such as anemia and leukopenia are more frequently seen in acute brucellosis cases. However, other hematological abnormalities such as severe thrombocytopenia, pancytopenia, acute hemolytic anemia, and disseminated intravascular coagulation are not infrequent.^5^ In the current study, out of 133 patients with fever lasting more than 5 days, 60 children were diagnosed as having acute brucellosis based on tube agglutination test method. The majority of patients (83%) declared raw animal milk/raw dairy products consumption while 45% had a positive family history of brucellosis. Al-Eissa, reported that, brucellosis in Saudi population presents in both sexes and in all ages, and that the main form of acquiring disease is through ingestion of raw milk and milk products obtained mainly from infected goats or camels, a traditional custom fostered by the nomadic heritage and dietary habits of the people.^1^ Patients with brucellosis usually present with fever, chills, malaise, weight loss, joint involvement, hepatosplenomegaly and lymphadenopathy.^5^ In the current study, the main symptoms at presentation in 60 children with brucellosis were excessive sweating (in 68%), bone aches (in 62%) and chills (in 55%). The main signs in these patients were arthritis/arthralgia (in 32%), and hepatomegaly (18%) and splenomegaly(15%). Hematological dyscrasia in study children with brucellosis included anemia (43%), leukopenia (38%), leukocytosis (20%) and pancytopenia (18%). These findings were in accordance with other reports conducted in both pediatric and adult patients with proven brucellosis.^7^,^13^,^14^ Similarly, in South-Western Saudi Arabia, Benjamin and Annobil, have reported an incidence of leucopenia in 38%, anemia in 64%, and thrombocytopenia in 28% of brucellosis candidates.^15^ Many other studies of hematological changes during the active course of brucellosis showed that leukopenia occurred in 33% of patients, anemia in 44%, thrombocytopenia in 5% and pancytopenia in 14%.^16^,^17^ Furthermore, Mantur BG detected pancytopenia in 10% of children suffering brucellosis.^14^ The relative frequency of pancytopenia with brucellosis varies from 3% to 21% in the previous studies, being relatively higher in adults than in children.^18^–^20^ The possible mechanisms suggested for pancytopenia include hypersplenism, granuloma formation in the BM, phagocytosis of formed elements by reticuloendothelial cells or BM depression due to the associated septicemia.^5^ Although anemia in brucellosis is expected to be due to BM involvement, numerous other pathogenetic mechanisms can be (and have been) implicated; Bourantas LK et al..^6^ reported that brucellosis induced an autoimmune process, culminating in autoimmune hemolysis. In this study, blood culture was positive for brucellosis in 23 children (21 for *B. melitensis* and 2 for *B.abortus*). BM culture was performed for 9 children, 3 of them were positive and had grown B. *melitensis*. The majority of children with brucellosis (63%) had serum agglutination titers of 1/160–1/320. In this study, the most common symptoms and signs, in 11 children with pancytopenia, included bone aches and weakness in (82%), sweating and chills in (64%), petechiea and purpura in (55%), hepatomegaly and splenomegaly (46%). The majority of children with pancytopenia (73%) have an agglutination titers of 1:320–1:640 or more. Furthermore, all cases with pancytopenia had positive blood culture, a findings which was nearly consistent with that obtained by other investigators.^2^,^4^,^5^,^8^,^14^,^20^,^21^

## Conclusion

In conclusion, despite pancytopenia seems to be an infrequent sequela of brucellosis in most of the literature, it was frequently seen in the current study. Thus, brucellosis should always be considered in the differential diagnosis of pancytopenia particularly in endemic areas as Saudi Arabia. Surveillance, testing and massive immunization of animals in endemic areas as well as an organized national brucellosis control program are prerequisites to eradicate the disease and hence complications.

## Figures and Tables

**Table 1:** Demographic and clinical characteristics in children with brucellosis.

Characteristic(s)	Number	Percent (%)

– Age (years)		
Mean ± SD	7.6±1.8	
Range	5–16	
– Males/females	43/17	71.7
– Duration of fever (days), X±SD	9.6±5.3	
– History of raw milk/dairy products consumption	50	83.3
– Family history of brucellosis	27	45

**Symptoms**		
– Excessive sweating	41	68.3
– Bone ache	37	61.7
– Chills	33	55
– Painful or swollen joints	22	36.7
– Weakness	21	35

**Signs**		
– Arthritis/arthralgia	19	32
– Hepatomegaly	11	18
– Splenomegaly	9	15
– Hepatosplenomegaly	4	7
– Lymphadenopathy	4	7
– Petechiae and purpura	3	5

**Table 2:** Hematological manifestations and microbiologic profile in children with brucellosis.

Presentation	Number	Percent (%)

**Hematological manifestations**		
– Anemia†	26	43.3
– Leucopenia††	23	38.3
– Leucocytosis‡	12	20.0
– Pancytopenia	11	18.3
– Lymphocytosis‡	5	8.3

**Cultures :**		
– Positive blood culture	23	38.3
– B.*melitensis*	21	35
– B.*abortus*	2	3.3
– Positive bone marrow culture*	3	5
– Negative bone marrow culture	6	10

**Agglutination titers**		
– Titers ≥1/160–1/320	38	63.3
– Titers >1/320–1/640 & more	22	36.7

* Positive for *B.melitensis*

† Defined as hemoglobin level and or red blood cells is below reference value for age and sex [22,23].

†† Defined as total leucocytic count is below reference value for age and sex [22,23].

‡ Defined as total leucocytic count/lymphocytic count is above reference value for age and sex [22,23].

**Table 3:** Clinical and laboratory findings in pancytopenia cases.

Finding	Number (11)	Percent (%)

**Symptoms**		
– Bone aches & weakness	9	81.8
– Sweating & chills	7	63.6
– Painful & swollen joints	5	45.5

**Signs**		
– Petechiae & purpura	6	54.5
– Hepatomegaly	5	45.5
– Splenomegaly	5	45.5
– Hepatosplenomegaly	3	27.3
– Arthritis/arthralgia	3	27.3
– Generalized lymphadenopathy	3	27.3

**CBC (X±SD)**		
– Hemoglobin (gram/dl)	5.9±2.8	
– WBC (cell × 10^9^/ml)	3.12±1.3	
– Platelets (cell × 10^9^/ml)	32.7±4.7	

**Agglutination titers, n(%):**		
– Titers ≥1/160–1/320	3	27.3
– Titers > 1/320–1/640 or more	8	72.7

Positive blood culture*	11	100

* positive for *B.melitensis*
